# Blood pressure, body mass index and risk of cardiovascular disease in Chinese men and women

**DOI:** 10.1186/1471-2458-10-189

**Published:** 2010-04-12

**Authors:** Hongwei Wang, Jie Cao, Jianxin Li, Jichun Chen, Xigui Wu, Xiufang Duan, Jianfeng Huang, Dongfeng Gu

**Affiliations:** 1 Department of Evidence Based Medicine, Cardiovascular Institute and FuWai Hospital, Chinese Academy of Medical Sciences and Peking Union Medical College, Beijing, China

## Abstract

**Background:**

It is still uncertain whether increased blood pressure (BP) has a stronger effect on the risk of cardiovascular disease (CVD) in lean persons than in obese persons. We tested it using a data set collected from a large cohort of Chinese adults.

**Methods:**

Systolic and diastolic BP, body mass index (BMI) and other variables were measured in 169,871 Chinese men and women ≥ 40 years of age in 1991 using standard protocols. Follow-up evaluation was conducted in 1999-2000, with a response rate of 93.4%. Data were analyzed with Cox proportional hazards models.

**Results:**

After adjusted for age, sex, cigarette smoking, alcohol consumption, high school education, physical inactivity, geographic region, and urbanization, we found that the effects of systolic or diastolic BP on risk of CVD generally increased with the increasing BMI levels (underweight, normal, overweight, and obese). For example, hazard ratios (HRs) and 95% confidence interval (CI) per 1- standard deviation (SD) increase in systolic BP within corresponding BMI levels were 1.27(1.21-1.33), 1.45(1.41-1.48), 1.52 (1.45-1.59) and 1.63 (1.51-1.76), respectively. Statistically significant interactions (P < 0.0001) were observed between systolic BP, diastolic BP and BMI in relation to CVD. In baseline hypertensive participants we found both obese men and women had higher risk of CVD than normal-weight persons. The multivariate-adjusted HRs(95%CI) were 1.23(1.03-1.47) and 1.20(1.02-1.40), respectively.

**Conclusion:**

Our study suggests that the magnitude of the association between BP and CVD generally increase with increasing BMI. Hypertension should not be regarded as a less serious risk factor in obese than in lean or normal-weight persons in Chinese adults.

## Background

Cardiovascular disease (CVD) was the leading cause of death in China in recent decades [1]. Prospective cohort studies have documented a strong, linear, and independent positive association between blood pressure (BP) levels and risk of CVD incidence and mortality among the general population [2-5]. Meanwhile, obesity has already been shown to play an important role for CVD [6]. As important risk factors of CVD, hypertension and obesity both showed increasing trends among Chinese population [7,8]. However, it is highly debatable on the role of obesity as an effect modifier in the association between BP and CVD. Some studies have reported that hypertension or elevated BP has a stronger effect on CVD risk in lean than in obese persons [9-15], whereas others either have reported no such relation or have even reported some opposite results [16-19]. Most previous studies were conducted in western populations in which the mean body mass index (BMI) was relatively high by comparison with China. In the present study, data from a large population-based prospective cohort in China were used to analyze the relationship of BP and BMI on risk of CVD.

## Methods

### Study population

In the 1991 China National Hypertension Survey [20], a multistage random cluster sampling design was used to select a representative sample of the general Chinese population aged 15 years and older from 30 provinces of mainland China. Of the 30 provinces, 13 were not included in the follow-up study because contact information was not available for study subjects. The baseline characteristics of subjects from the remaining 17 provinces that were included in this analysis were similar to those in the 13 excluded provinces [21]. In 1999 and 2000, from the 17 provinces, 169871 study subjects (83,533 men and 86,338 women) who were 40 years or older at their baseline examination were eligible for participation in the follow-up study. From them, a total of 158, 666 (93.4%) study participants (or their proxies) were identified and interviewed as part of the follow-up study. In this report, study participants with missing BP values (n = 285), a history of CVD (n = 4,192) and missing BMI values (n = 14721) at the baseline examination were excluded from related analyses, respectively.

### Baseline examination

All baseline data were collected at a single clinic visit by specially trained physicians and nurses using standardized methods with stringent levels of quality control [20]. Data on demographic characteristics, medical history, and lifestyle risk factors were obtained using a standard questionnaire administered by trained staff. Work-related physical activity was assessed because leisure-time physical activity was uncommon. Cigarette smokers were defined as having smoked at least one cigarette per day for 1 or more years. The amount and type of alcohol consumed during the past year were collected. Three BP measurements were taken after the study participant had been seated quietly for 5 minutes using a standard mercury sphygmomanometer according to a standard protocol [22]. The first and fifth Korotkoff sounds were recorded as systolic and diastolic BP, respectively. Participants were instructed not to eat, drink alcohol, coffee, or tea, smoke, or exercise for at least 30 minutes prior to their BP measurement. The mean of 3 BP measures was used in all analyses. Body weight and height were measured in light indoor clothing without shoes using a standard protocol. BMI was calculated as weight in kilograms divided by height in square meters. According to the definition of obesity from Working Group on Obesity in China (WGOC) [23], participants in the present study were divided into 4 BMI levels including underweight (<18.5 kg/m^2^), normal (18.5-23.9 kg/m^2^), overweight (24-27.9 kg/m2), and obese (≥ 28 kg/m2). Baseline hypertensive participants were defined as participants with systolic BP ≥ 140 mmHg or diastolic BP ≥ 90 mmHg, or having taken antihypertensive medication in last two weeks before baseline interview.

### Follow-up data collection

The follow-up examination was conducted between 1999 and 2000, which included tracking study participants or their proxies to a current address, performing in-depth interviews with the participants or proxies to ascertain disease status and vital information, and obtaining hospital records and death certificates. If a study participant reported a hospitalization or emergency room overnight-stay due to acute myocardial infarction or stroke during the in-person interview, the participant's hospital records, including medical history, physical examination findings, laboratory test results, and discharge diagnosis, were abstracted by trained staff using a standard form. In addition, photocopies of selected sections of the participant's in-patient record, discharge summary, electrocardiogram, and pathology reports were obtained. All deaths reported during the in-person interview were verified by obtaining death certificates from the local public health department or police department. If death occurred during a hospitalization, the participant's hospital records and autopsy results were also abstracted by trained staff using a standard form. If death occurred outside of the hospital, detailed information on medical history was obtained from a family member or healthcare provider.

An end-point assessment committee within each province reviewed all abstracted information to confirm or reject the occurrence of study outcomes using pre-established criteria. A study-wide end-point assessment committee at the Chinese Academy of Medical Sciences in Beijing, China, reviewed all medical records and determined the final diagnosis of the event or the underlying cause of death. Two committee members independently verified the diagnosis and discrepancies were adjudicated by discussion involving additional committee members. All members of the local and study-wide end-point assessment committees were blinded to the study participant's baseline risk factor information. Causes of death were coded according to the International Classification of Diseases, Ninth Revision (ICD-9).

For this analysis, the CVD group lists those with a confirmed diagnosis of acute myocardial infarction or stroke during the follow-up period or mortality with a cardiovascular event (ICD-9 390.0-398.9, 401.0-429.9, and 430.0-438.9) listed as an underlying cause of death. The coronary heart disease (CHD) group lists those with a confirmed diagnosis of acute myocardial infarction during the follow-up period or CHD listed as an underlying cause of death (ICD-9 410.0-414.9). Finally, the stroke group lists those with a confirmed diagnosis of stroke during the follow-up period or stroke listed as an underlying cause of death (ICD-9 430.0-438.9).

This study was approved by the Cardiovascular Institute and Fu Wai Hospital Ethics Committee and the Tulane University Health Sciences Center Institutional Review Board. Written informed consent was obtained from all study participants at their follow-up visit.

### Statistical analysis

Previous study found that there was a linear and independent relationship between systolic and diastolic BP and the risk of CVD in our cohort [5]. Thereafter, all analyses were performed with systolic BP and diastolic BP on a continuous scale. Data were analyzed with Cox proportional hazards models. We analyzed the modifying effect of BMI on the association between BP and CVD by computing the HRs for a 1- standard deviation (SD) increase in systolic BP and diastolic BP by fitting a separate model for each of the BMI levels. In the present study, SD of systolic BP and diastolic BP were 22.1 mmHg and 12.0 mmHg, respectively.

We adjusted for baseline age, sex, cigarette smoking, alcohol consumption, high school education, physical inactivity, geographic region (south vs. north), and urbanization (rural vs. urban) in the models. Interactions between BP and BMI were then modeled by incorporating an interaction term between systolic BP or diastolic BP and BMI.

All statistical models were performed with the SAS statistical software (version 9.1; SAS Institute, Cary, NC).

## Results

Baseline characteristics of study participants according to BMI levels are presented in Table 1. Those with higher BMI levels were most likely to be younger, female, not alcohol drinkers or cigarette smokers, living in urban or north China, physically inactive, having high school education and having higher systolic BP or diastolic BP at the baseline examination. The obese and underweight participants both had higher crude incidence of CVD than normal-weight persons.

**Table 1:** Baseline Characteristics and CVD Incidence Rates at Follow-Up According to Baseline BMI

Baseline characteristics	Baseline BMI, kg/m^2^				P value for trend
		
	<18.5	18.5-23.9	24-27.9	≥ 28	
No. of participants	16,543	79,220	33,401	10,404	--
Systolic BP (mean (SD), mm Hg)	121.8(22.8)	123.6(21.2)	130.5(21.8)	137.8(23.1)	<0.0001
Diastolic BP (mean (SD), mm Hg)	72.5(11.7)	75.9(11.4)	80.9(11.7)	84.6(12.2)	<0.0001
BMI (mean (SD), kg/m^2^)	17.2(1.1)	21.7(1.8)	26.9(1.3)	32.6(4.0)	<0.0001
Person-years of follow-up	129,791	633,410	265,983	819,94	--
No. of CVD events	1,725	5,134	2,511	1046	--
Incidence of CVD, per 100,000 Person-years	1329.06	810.53	944.05.19	1275.70	NA
Age (mean (SD), year)	60.1(11.5)	54.7(10.5)	54.4(9.8)	56.1(9.5)	<0.0001
Men (%)	45.4	52.2	47.8	36.0	<0.0001
High school education (%)	11.1	21.0	32.9	28.6	<0.0001
Cigarette smokers (%)	44.0	40.8	33.8	26.9	<0.0001
Alcohol consumption (%)	17.4	21.9	19.1	14.5	<0.0001
Physical inactivity (%)	31.7	31.9	42.4	44.8	<0.0001
North (%)	41.3	60.8	77.1	85.0	<0.0001
Urban (%)	35.4	51.5	78.8	84.7	<0.0001
Hypertension(%)	20.2	21.8	35.0	49.9	<0.0001
Hypertension treatment(%)	0.9	1.5	3.9	7.0	<0.0001

SD, standard deviation; CVD, cardiovascular disease; BMI, body mass index; BP, blood pressure; NA: not applicable.

Table 2 presents the results for the associations of BP with incident CVD (fatal and nonfatal cases together) by separate baseline BMI levels and for all levels combined. Both systolic BP and diastolic BP were strongly associated with CVD with statistical significance at each BMI level and at all levels combined after adjusted for age, sex, cigarette smoking, alcohol consumption, high school education, physical inactivity, geographic region, and urbanization. HRs and 95% CI per 1-SD increase in systolic BP and diastolic BP computed with Cox proportional hazards models for all BMI levels combined were 1.42 (1.40-1.45), and 1.40 (1.37-1.43). HRs and 95%CI per 1-SD increase in systolic BP or diastolic BP both increased among four BMI levels (underweight, normal, overweight, and obese). The results indicated that the effects of BP on risk of CVD generally increased with the increasing BMI levels.

**Table 2:** HRs on Risk of CVD for Relation of a 1-SD Increase in Systolic BP and Diastolic BP According to Baseline BMI

BMI	Systolic BP		Diastolic BP	
	
	HR*	95%CI	HR*	95%CI
<18.5 kg/m^2^	1.27	1.21-1.33	1.25	1.19-1.31
18.5-23.9 kg/m^2^	1.45	1.41-1.48	1.42	1.38-1.46
24-27.9 kg/m^2^	1.52	1.45-1.59	1.49	1.41-1.56
≥ 28 kg/m^2^	1.63	1.51-1.76	1.53	1.41-1.65
All	1.42	1.40-1.45	1.40	1.37-1.43
P for interaction *	<0.0001		<0.0001	

HRs, hazard ratios; CI, confidence interval; CVD, cardiovascular disease; BMI, body mass index; BP, blood pressure.

*Adjusted for age, sex, cigarette smoking, alcohol consumption, high school education, physical inactivity, geographic region (south vs. north), and urbanization (rural vs. urban).

Statistical evidence was also found for interactions between BMI and both systolic BP and diastolic BP in the prediction of all CVD (both P < 0.0001). When participants were divided by sex (men vs. women), the interactions between BMI and both systolic BP and diastolic BP in the prediction of all CVD remained positive (both P < 0.001) (Table 3).

**Table 3:** HRs and 95%CI for the Relation of a 1-SD Increase in Systolic BP and Diastolic BP According to Baseline BMI Divided by Sex (men and women)

BMI	Systolic BP		Diastolic BP	
	
	HR*	95%CI	HR*	95%CI
Men				
<18.5 kg/m^2^	1.26	1.18-1.35	1.25	1.17-1.33
18.5-23.9 kg/m^2^	1.48	1.42-1.53	1.46	1.41-1.52
24-27.9 kg/m^2^	1.63	1.53-1.74	1.48	1.38-1.58
≥ 28 kg/m^2^	1.79	1.54-2.07	1.57	1.36-1.80
All	1.47	1.43-1.50	1.42	1.38-1.46
P for interaction*	<0.0001		<0.0001	
Women				
<18.5 kg/m^2^	1.27	1.18-1.35	1.25	1.16-1.34
18.5-23.9 kg/m^2^	1.41	1.35-1.47	1.37	1.31-1.43
24-27.9 kg/m^2^	1.43	1.34-1.52	1.49	1.39-1.60
≥ 28 kg/m^2^	1.58	1.44-1.74	1.51	1.37-1.66
All	1.38	1.34-1.42	1.37	1.33-1.41
P for interaction*	0.0007		0.0008	

HRs, hazard ratios; CI, confidence interval; CVD, cardiovascular disease; BMI, body mass index; BP, blood pressure.

*Adjusted for age, cigarette smoking, alcohol consumption, high school education, physical inactivity, geographic region and urbanization.

**Figure 1:**
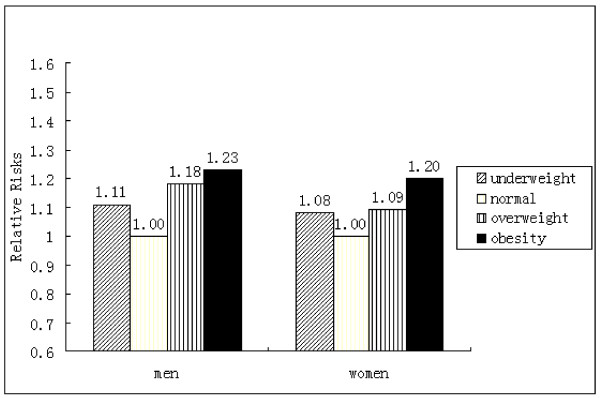
**Relative Risks of CVD According to Baseline BMI in Hypertensive Participants (men and women)**. Participants with the lowest BP and BMI between 18.5 and 23.9 kg/m^2 ^was used as the reference group (RR 1.00).

We examined the association between levels of BMI and risk of CVD in baseline hypertensive participants after adjusted multivariate factors by Cox proportional hazards models (Figure 1). Compared with the reference group (BMI between 18.5 and 23.9 kg/m^2^), among four corresponding BMI levels men with BMI of 28 kg/m^2 ^or higher had the highest hazard ratio of CVD, 1.23(95% CI, 1.03-1.47). For women, participants with BMI of 28 kg/m^2 ^or higher also had the highest hazard ratio, 1.20(95% CI, 1.02-1.40).

**Figure 2:**
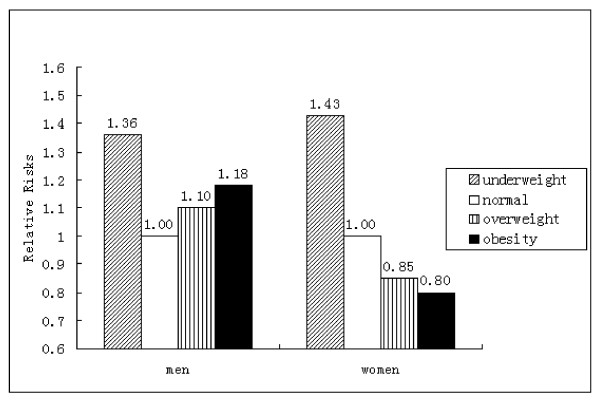
**Relative Risks of CVD According to Baseline BMI in Non-hypertensive Participants (men and women)**. Participants with the lowest BP and BMI between 18.5 and 23.9 kg/m^2 ^was used as the reference group (RR 1.00).

We also examined the association between levels of BMI and risk of CVD in baseline non-hypertensive participants (Figure 2). On the contrary, we found that both men and women with BMI of 18.5 kg/m^2 ^or lower had the highest risk of CVD among four corresponding BMI levels. Compared with the reference group (BMI between 18.5 and 23.9 kg/m^2^), the multivariate-adjusted HRs(95%CI) were 1.36(1.22-1.52) and 1.43(1.25-1.63), respectively.

We also examined the interactions between BP and western BMI groupings including underweight (<18.5 kg/m^2^), normal (18.5-24.9 kg/m^2^), overweight (25-29.9 kg/m^2^), and obese (≥ 30 kg/m^2^) in our cohort study, and the pattern and conclusion of results did not change.

### Sensitivity Analysis

After excluding the incident CVD that occurred during the first 3 years of follow-up, we still found that the effects of BP on risk of CVD generally increased with the increasing BMI levels. HRs and 95% CI per 1-SD increase in systolic BP computed with Cox proportional hazards models within four BMI levels (underweight, normal, overweight, and obese) were 1.26(1.19-1.33), 1.44(1.40-1.49), 1.51(1.43-1.58) and 1.61(1.47-1.76), respectively. HRs and 95% CI per 1-SD increase in diastolic BP within four BMI levels were 1.25(1.18-1.32), 1.40(1.36-1.45), 1.46(1.38-1.54) and 1.48(1.35-1.63), respectively. Statistical evidence was still found for interactions between BMI and both systolic BP and diastolic BP in the prediction of all CVD (both P < 0.001).

## Discussion

In the present large prospective cohort of Chinese adults, our study observed the significant interactions between systolic BP or diastolic BP and BMI in relation to CVD (both P < 0.0001) after other important risk factors were adjusted, including age, sex, cigarette smoking, alcohol consumption, high school education, physical inactivity, geographic region and urbanization. Furthermore, we found the interactions between systolic BP or diastolic BP and BMI in relation to CVD remained positive when participants were divided by men and women (both P < 0.001). These findings suggested that the association between BP and CVD increased with increasing BMI in Chinese population.

Findings of interaction analysis (table 2) suggested that the effects of BP on risk of CVD generally increased with the increasing BMI levels, for instance, HRs per 1-SD increase in systolic BP within 4 BMI levels (underweight, normal, overweight, obese) were 1.27, 1.45, 1.52 and 1.63, respectively, which indicated that elevated BP would be more harmful in obese persons for CVD risk. Therefore, obese persons would have more benefits from lowering the BP. Considering that hypertension has become the leading preventable risk factor for CVD and all-cause mortality in developing countries [24,25], our observation may have important implications for interpreting the benefit of the treatment of hypertension.

Relationship of BP and BMI on the risk of CVD has not a universal conclusion. High risk among lean hypertensive persons has been reported for CVD risk [9-15] and total mortality [26,27]. On the contrary some previous studies [16-19] did not support the suggestion that lean persons were more dangerous than obese persons on risk of CVD. In the 26 year follow up and the 34 year follow up in Framingham [17] it was reported that hypertension is at least as dangerous in obese as in lean persons at all ages in either sex. Stamler and coauthors [14] reported that lean hypertensive persons had a higher risk not only for death of CVD, but also for death due to cirrhosis, nonmalignant respiratory disease, violence, and malignant neoplasms than obese hypertensive persons. This suggested that excess alcohol intake and smoking were important factors that contributed to the excess risk. However, in a recent study, Karri Silventoinen found that the adjustment for socioeconomic factors had no effect on the interactions between BP and BMI and meanwhile observed a stronger effect of BP on CVD risk in obese as opposed to lean or normal-weight men in a large cohort of young Swedish men [19]. Our results confirmed the findings from Karri Silventoinen with adjustment for alcohol consumption, cigarette smoking and other important risk factors of CVD. We found significant interactions between systolic BP, diastolic BP and BMI in relation to CVD in Chinese men and women. Indeed, we observed that the effects of BP on CVD risk generally increased with the increasing BMI levels.

In the present study, in baseline hypertensive participants obese men and women had the highest HRs of CVD among 4 BMI levels (Figure 1). Our results did not support that hypertension or high blood pressure was a less serious risk factor for CVD in obese than in lean or normal-weight persons in Chinese population.

It is very interesting that in baseline non-hypertensive participants we found lean men and women had the highest HRsof CVD among 4 BMI levels (figure 2). An explanation for these different findings between hypertensive participants and non-hypertensive participants is the change of total peripheral resistance during the pathologic process of hypertension. Raised total peripheral vascular resistance is the hallmark of essential hypertension and the major determinant of systemic hypertensive vascular disease [28]. Arterial BP is determined by cardiac output and total peripheral resistance. Any factor which raises cardiac output or total peripheral resistance will raise BP. Because obese persons are usually associated with the augmented cardiac output (in response to elevated metabolic requirements), at a given level of low or normal BP, obese persons have low total peripheral resistance while lean persons have high total peripheral resistance which result in high risk of CVD. But it changed when BP increases continuously. As hypertension becomes established, total peripheral resistance increases and cardiac output reverts to normal [29]. Thus, at a given level of high BP, total peripheral resistance is nearly the same in an obese person comparing a lean or normal-weight one. However, obese persons always were associated with other factors such as inflammation-sensitive plasma proteins [30] which may induce excess risk of CVD. As a consequence, at low or normal BP levels lean persons have higher risk of CVD than normal-weight or obese persons, but at high BP levels obese persons have higher risk than lean or normal-weight persons. The mechanism of combination effect of hypertension and obesity needs further researches.

Our study has many advantages. It was conducted in a large, nationally representative sample of the general population, had a very high follow-up rate, and used stringent quality control procedures in assessing baseline variables and clinical study outcomes. In addition, the study population had a relatively low average but correspondingly wide range of BP levels. This allowed us to examine the risk of CVD stratified by baseline BMI at a lower level of BP. However, our study has potential limitations as well. Some important risk factors for CVD, such as serum lipids, diet, and leisure-time physical activity were not measured and thus we cannot fully test whether adjustment of the HRs for these risk factors would change the interactive effect between BP and BMI. Variables including HDL cholesterol, LDL cholesterol and blood glucose would be possible confounders and adjustment for these variables might reduce the predictive effect of BMI on risk of CVD. However, major population-based studies, including the Framingham heart study [31], the Nurses' Health Study [32], and the US Male Health Professionals Study [33], have demonstrated that BMI is still an important risk factor for CHD incidence even after adjustment for age, hypertension, diabetes, smoking status, and cholesterol levels.

## Conclusions

In the present large, nationally cohort study of Chinese men and women, we found that there are significant interactions between systolic BP, diastolic BP and BMI in relation to CVD. The effects of BP on CVD risk generally increased with the increasing BMI levels. Our results did not supported that high blood pressure would be less harmful in obese than in lean or normal-weight persons.

## Competing interests

The authors declare that they have no competing interests.

## Authors' contributions

HW did the analysis of the data and made the draft of the manuscript. JC, JL, JC, XW, XD and JH conducted the investigation and participated in disscussing. DG supervised the project and revised the manuscript. All authors read and approved the final manuscript.

## Pre-publication history

The pre-publication history for this paper can be accessed here:

http://www.biomedcentral.com/1471-2458/10/189/prepub
